# New York College of Veterinary Surgeons

**Published:** 1887-04

**Authors:** 


### NEW YORK COLLEGE OF VETERINARY SURGEONS.

The thirtieth annual Commencement of the New York College of Veterinary Surgeons and School of Comparative Medicine was held at the Carnegie Laboratory. The graduating class consisted of Donald E. Watson, James McKee, Samuel Atchison and Joseph Blumental. Prof. Joseph H. Raymond, M. D., of Brooklyn, told them to what an important calling they had elevated themselves, and the degrees were conferred by President W. T. White. The valedictory was delivered by Samuel Atchison. Prizes were awarded as follows: Faculty prize for best general examination, gold medal, to James McKee; McLane prize for best junior examination, silver medal, to L. E. Simpkins; Comstock prize for best examination in anatomy, a pocket case of veterinary instruments, to Donald E. Watson; Jenkins prize for second best junior examination, $10 and a veterinary book, to A. Jerome; Hamil prize for second best senior examination, gold medal, to Samuel Atchison.

